# A Case of Heterotopic Gastric Tissue in Duodenal Bulb: An Interesting Endoscopic Finding

**DOI:** 10.7759/cureus.24271

**Published:** 2022-04-19

**Authors:** Sohaib Khan, Saleha Niaz, Mudassir Khan, Rajiv Singh, Stephanie R Murphy

**Affiliations:** 1 Internal Medicine, Parkview Medical Center, Pueblo, USA; 2 Internal Medicine, Nawaz Sharif Medical College, Gujrat, PAK; 3 Internal Medicine, Dow University of Health Sciences, Civil Hospital Karachi, Karachi, PAK; 4 Gastroenterology, Parkview Medical Center, Pueblo, USA

**Keywords:** metaplasia, polyp, duodenal bulb, duodenum, heterotopia, gastric

## Abstract

Gastric heterotopia (GH) is a rare, congenital condition where gastric tissue is found outside of its normal location in the gastric mucosa. It is usually benign and can be found throughout the gastrointestinal (GI) tract. In the duodenum, it is usually seen as multiple polyps, specifically in the duodenal bulb. Here, we discuss the case of a 67-year-old male patient who presented with hematemesis, melena, and abdominal pain. Esophagogastroduodenoscopy (EGD) and biopsy revealed a mass consisting of heterotopic gastric mucosa along with an esophageal ulcer. In this article, we will discuss the literature related to the clinical presentation, diagnosis, and management of GH.

## Introduction

The word heterotopia is derived from the Greek language, which refers to tissues that are found in an unusual place [1]. Gastric heterotopia (GH) is an uncommon, benign, congenital condition characterized by the presence of gastric tissue outside of the stomach [2,3]. It can be found throughout the gastrointestinal (GI) tract and usually occurs in the form of polyps, nodules, or tumorous growths [1-3]. In the duodenum, GH is most commonly seen in the duodenal bulb in the form of multiple polyps in approximately 0.5 to 2% of the general population [2,4]. It has characteristic endoscopic and pathologic findings that are often mistaken for gastric metaplasia [3]. Although GH is usually asymptomatic, it can sometimes lead to abdominal pain, nausea, vomiting, and GI bleeds [5]. Here, we present a 67-year-old male who was incidentally found to have a heterotopic gastric mass.

## Case presentation

**Figure 1:**
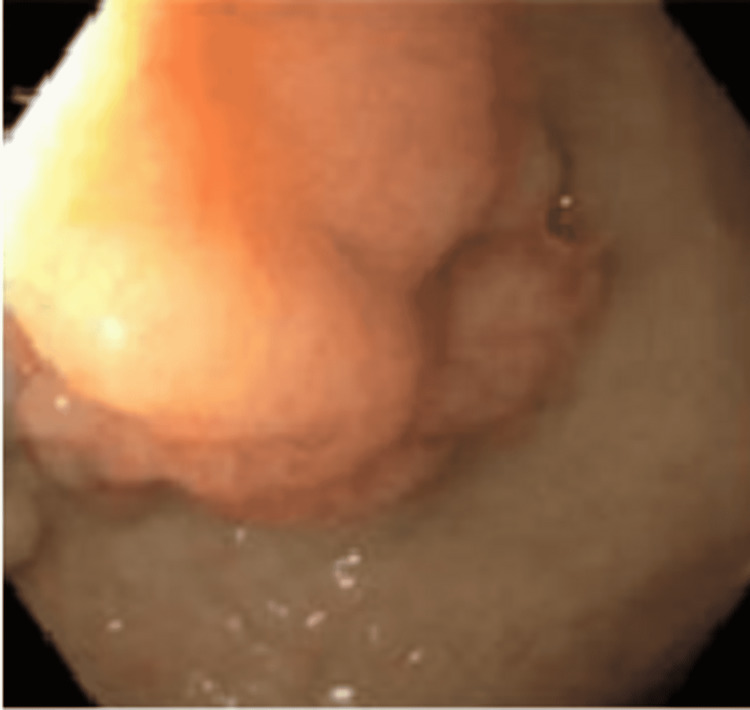
EGD showing a 1.5 cm non-ulcerated and non-bleeding mass in the duodenal bulb. EGD: esophagogastroduodenoscopy.

**Figure 2:**
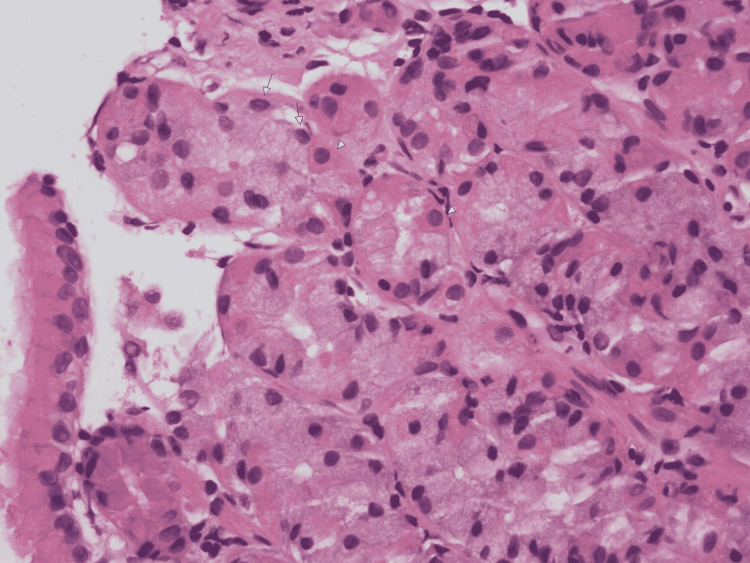
H&E stain at 400× magnification showing fundic glands containing chief cells (arrows) and parietal cells (arrowheads). H&E: hematoxylin and eosin.

**Figure 3:**
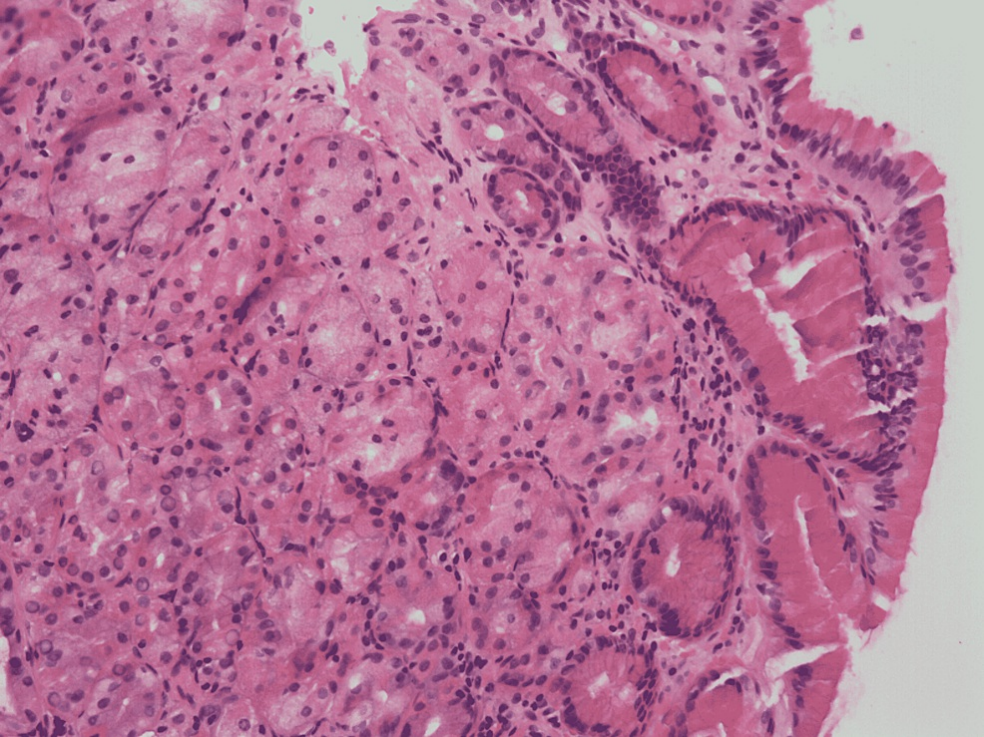
H&E stain at 200× magnification showing gastric fundic-type mucosa, with mucinous foveolar epithelium (right-side of image) overlying closely packed glands containing chief and parietal cells. H&E: hematoxylin and eosin.

A 67-year-old male patient was admitted with multiple episodes of melena and hematemesis that started three days prior to admission and was associated with nausea, abdominal pain, and diarrhea. Physical examination showed mild diffuse abdominal tenderness without guarding, rigidity, or rebound tenderness. Labs were significant for hemoglobin 7.7, hematocrit 22.5, creatinine 7.25, and blood urea nitrogen 143. A computerized tomography (CT) abdomen and pelvis showed fluid-filled distension of the stomach, cholelithiasis, colonic diverticulosis, and bilateral hydroureteronephrosis. Initial management included intravenous (IV) fluids, IV proton pump inhibitors (PPIs), placing a Foley catheter (Bard Medical Division, C. R. Bard, New Jersey, USA), and close hemodynamic monitoring. A subsequent esophagogastroduodenoscopy (EGD) showed an ulcer in the lower third of the esophagus along with endoscopic findings of suggestive intestinal metaplasia, an esophageal hiatal hernia, and a 1.5 cm mass in the duodenal bulb (Figure 1). Biopsy of the mass was consistent with benign gastric heterotopia (Figures 2, 3). The patient was started on a clear liquid diet with advancement as he tolerated. With the resolution of gastrointestinal bleeding and improvement of his condition, PPI was switched from IV to oral and the patient was discharged with a recommendation to follow-up with gastroenterology in an outpatient clinic.

## Discussion

Gastric heterotopia (GH) is defined as the presence of mature gastric tissue outside the gastric mucosa [2]. It can be found anywhere in the gastrointestinal (GI) tract [6]. GH in the duodenum is a benign condition and the incidence is reported to vary from 0.5 to 14%, although in the duodenal bulb, it is seen in only 0.5 to 2% of the general population [3,4]. Initial cases in the duodenum were identified on autopsies in a case series in 1927 [7]. GH is mainly congenital, with the underlying mechanism proposed as a defect in the differentiation of primitive endoderm stem cells that leads to the ectopic presence of gastric mucosa in the GI tract [3]. However, it has also been proposed that repeated injury to the duodenal mucosa by gastric acid can also lead to the development of fundic-type mucosa, making it an acquired process [2]. Narang et al. described an important histological differentiation between GH and gastric metaplasia, emphasizing that GH is purely a congenital entity [5]. In our patient’s case, biopsy-proven gastric heterotopia along with no prior history of acid-reflux symptoms favors the congenital origin of the mass, although his age, finding of esophageal ulcer and esophageal intestinal metaplasia cannot rule out chronic irritation of the duodenal mucosa.

GH is usually discovered as an incidental finding on endoscopy. Most of the patients are asymptomatic, but symptoms such as dyspepsia, heartburn, abdominal pain, obstruction, or GI bleeding have been attributed based on the location of heterotopic tissue [8]. Our patient’s presentation with GI bleed is most likely secondary to his esophageal ulcer disease since his duodenal GH had no stigmata of recent or active bleeding on endoscopy.

Diagnosis of GH is made by a combination of the clinical picture, radiologic imaging, endoscopic, and histopathological evaluation. Treatment includes endoscopic or surgical resection to prevent complications [9]. As most cases of GH are benign, extensive heterotopia increases the potential risk for adverse outcomes, including ulceration, stricture, massive GI bleeding, or rarely death [3]. Rare incidences of dysplasia and adenocarcinoma with GH have also been reported in the literature [8]. Due to these reasons, prompt identification of GH is significant. Further studies are needed to assess the role of routine surveillance for GH.

## Conclusions

Gastric heterotopia (GH) is the presence of gastric tissue outside of the stomach and can be found anywhere throughout the gastrointestinal (GI) tract, although it is very uncommon in the duodenal bulb. It is mostly congenital, but repeated exposure to gastric acid can also lead to its development. Like our patient, most cases of GH are incidentally discovered, but sometimes it can cause ulceration and massive GI bleeding, leading to significant morbidity and mortality. Due to these reasons, prompt identification of GH is significant. Further studies are also needed to assess the role of routine surveillance for GH.
